# Recent Advances in Understanding LPMO Catalysis

**DOI:** 10.1021/acs.biochem.3c00458

**Published:** 2023-11-02

**Authors:** Lukas Rieder, Morten Sørlie

**Affiliations:** † Institute of Molecular Biotechnology, Graz University of Technology, Petersgasse 14, A-8010 Graz, Austria; ‡ Faculty of Chemistry, Biotechnology and Food Science, Norwegian University of Life Sciences (NMBU), Chr. M. Falsensvei 18, N-1432 Ås, Norway

Lytic polysaccharide monooxygenases (LPMOs) are monocopper enzymes involved in the degradation of recalcitrant polysaccharides such as chitin and cellulose. LPMOs are classified as auxiliary active (AA) enzymes and categorized into eight families (AA9–AA11 and AA13–AA17) within the CAZy database that categorizes structurally related LPMOs based on sequences, which typically are linked to their origin, i.e., mainly bacterial or fungal. Central to catalysis is the activation of H_2_O_2_ by Cu(I) in the active side for a controlled C–H bond hydroxylation followed by the dissociation of a glycosidic bond in a peroxygenase reaction. It is intriguing that a monocopper enzyme can harness such oxidative power, and this serves as an inspiration to understand the underlying catalytic mechanism of LPMOs.

**Figure 1:**
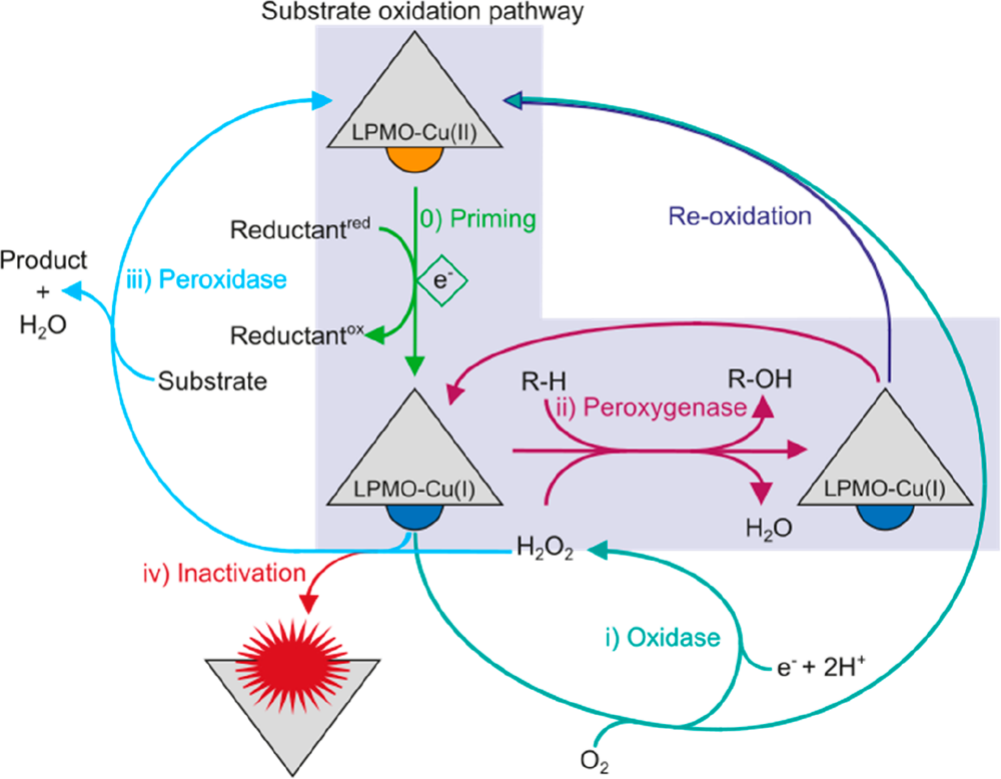
Schematic illustration of the interconnected reaction pathways of LPMOs. Central to the LPMO reaction is the one-electron reduction from the resting Cu(II) to the active Cu(I) state known as priming (0). Subsequently, four different pathways are possible depending on the reaction conditions. In the absence of a carbohydrate substrate, O_2_ will be reduced to H_2_O_2_ in an oxidase reaction (i). In the presence of a carbohydrate substrate and H_2_O_2_, LPMOs catalyze a peroxygenase reaction resulting in the release of oxidized products (ii). In addition, LPMOs catalyze the oxidation of their reductant in a peroxidase reaction (iii) or inactivate due to autocatalytic oxidation of the active site (iv).

The reduction of the resting state Cu(II) to Cu(I) can be achieved by a plethora of small molecules as well as other enzymes. Once reduced, several reaction pathways can occur depending on the reaction conditions (Figure 1). (i) In the absence of a carbohydrate substrate, O_2_ is reduced, with a concomitant depletion of the reductant, to H_2_O_2_ (oxidase activity). (ii) In the presence of a carbohydrate substrate and H_2_O_2_, oxidative cleavage of the polymer occurs (peroxygenase activity), and only priming amounts of the reductant are needed. For both conditions, two other pathways exist: (iii) LPMO-catalyzed oxidation of the reductant (peroxidase activity) and (iv) inactivation through oxidative damage to the enzyme. All four pathways depend on the nature of the LPMO based upon its AA family, as this significantly affects the kinetics of the different pathways. Moreover, the nature of the reductant affects all pathways. In sum, this shows the complexity of studying the LPMO mechanism.

**Figure 2:**
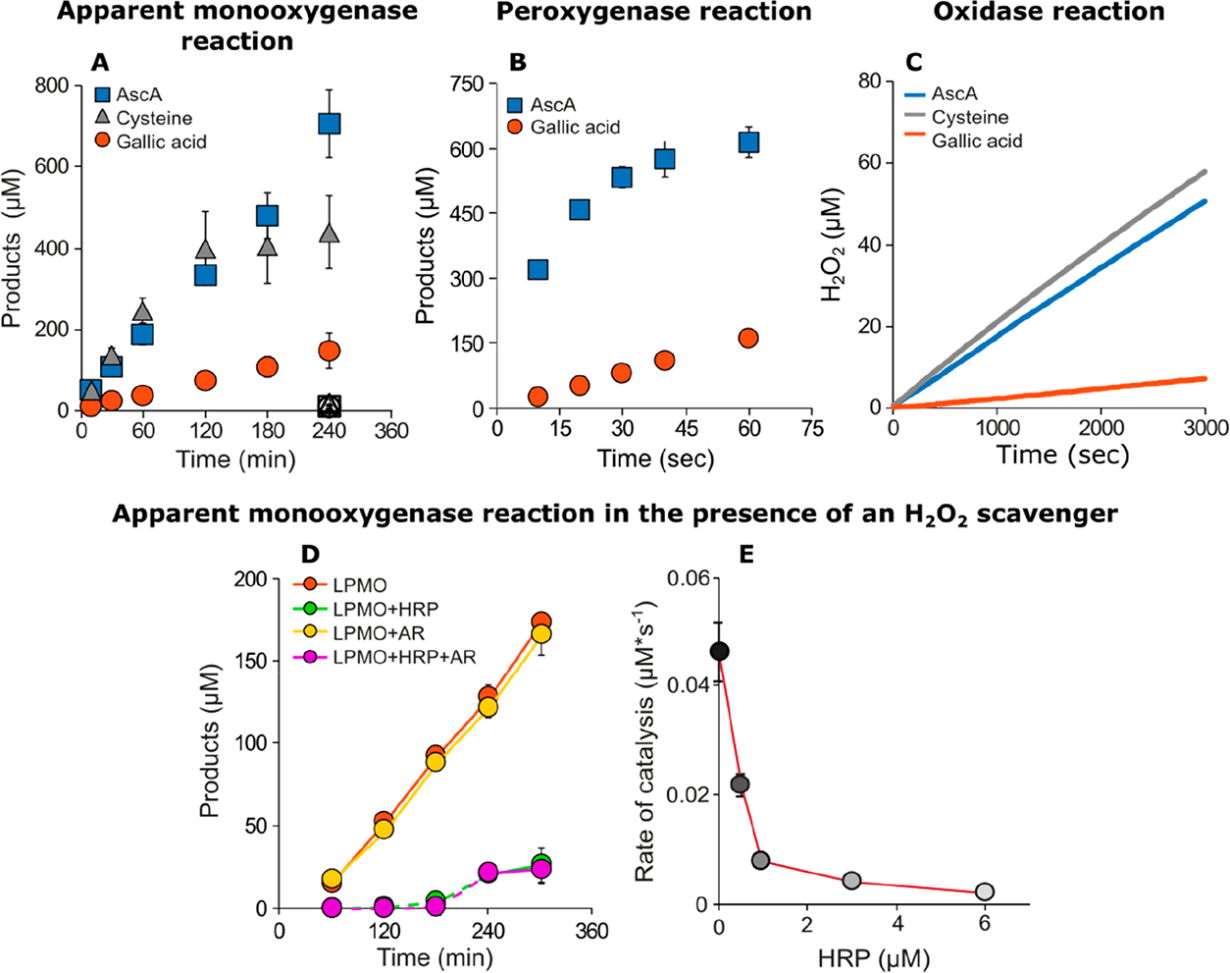
Progress curves showing the formation of oxidized products (A, B, D, E) or H_2_O_2_ (C) using *Nc*AA9C (A–D) or *Af*AA11B (E) as the catalyst. Note the difference in the time scale when comparing monooxygenase and peroxygenase reactions (A vs B) and that H_2_O_2_ production and product formation follow the same reductant-dependent trend (A vs C). For exact reaction conditions, please have a look at the original literature. Reproduced from refs (2) and (3). Copyright [2021] The Authors.

One central question has been whether the mechanism catalyzing C–H bond cleavage is of monooxygenase (R–H + O_2_ + 2e^–^ + 2H^+^ → R–OH + H_2_O) or peroxygenase (R–H + H_2_O_2_ → R–OH + H_2_O) nature (Figure 2A,B). Stepnov et al. showed for AA10_07 (AA10, bacterial) that the rate of reaction is independent of the LPMO concentration but rather dependent on the intrinsic *in situ* production of H_2_O_2_ caused by the LPMO oxidase activity, auto-oxidation of the external electron donor, and the presence of free Cu(II) in the samples under so-called monooxygenase conditions (atmospheric O_2_ and 1 mM external reductant).^1^ The observed rate of substrate oxidation was dependent on the nature and concentration of the reductant. Taking the advantage that certain LPMOs are active on soluble substrates, Rieder et al. showed that LPMO catalysis was completely quenched in the presence of the H_2_O_2_ scavenging enzyme horseradish peroxidase (HRP) under so-called monooxygenase conditions for *Nc*AA9C (*Neurospora crassa*, AA9, fungal) and *Af*AA11B (*Aspergillus fumigatus*, AA11, fungal), respectively (Figure 2D,E).^2,3^ Combined, the three studies showed that AA10s mainly depend on the auto-oxidation of the reductant while the AA9 and AA11 rest on the intrinsic oxidase activity for the *in situ* H_2_O_2_ production under so-called monooxygenase conditions (Figure 2A,C). Furthermore, AA11-type LPMOs have the highest oxidase activity followed by AA9 and then AA10, which seems to be connected to the reduction potential of the enzymes.^3^

Looking at the substrate oxidation, interestingly, *Nc*AA9C displayed a 2000-fold increase in the catalytic rate in the presence of H_2_O_2_ compared to O_2_ with the soluble cellopentaose as the substrate, while *Af*AA11B yielded an 80-fold increase for the soluble chitotetraose.^2,3^ Moreover, for *Nc*AA9C, a clear reductant effect on the peroxygenase reaction was observed as the apparent rate constant was 70 s^–1^ using ascorbic acid, 25 s^–1^ with gallic acid, and 6 s^–1^ employing cysteine. Nevertheless, once reaction conditions were met, *Nc*AA9C showed linear time course plots for concentrations of H_2_O_2_ up to 600 μM, and a Michaelis–Menten analysis yielded a *k*_cat_/*K*_m_ of 5.9·10^4^ M^–1^ s^–1^ for H_2_O_2_.

Quantifying peroxidase activity and the reductant effect has been developed through the determination of the half-saturating constants *K*_*m*R_^app^, a Michaelis–Menten type plot of rate vs. reductant concentration. Obtained values may be interpreted as such that a high value equals high peroxidase activity in that the reductant participates in futile turnover through the oxidation of the reductant. For *Af*AA11B catalyzed oxidation of chitotetraose in the presence of ascorbic acid, a value of 500 μM was determined.^3^

It is known that, in the presence of H_2_O_2_ and especially in the absence of substrate, the peroxidase reaction of LPMOs may lead to inactivation due to oxidative damage of the enzyme at the active site. A simple but illustrative monitoring of this is achieved by determining initial rates with increasing concentrations of reductant or H_2_O_2_. In a comparative study by Rieder et al. with *Nc*AA9C and *Ls*AA9A (*Lentinus similis*, AA9, fungal), the latter appeared to experience inactivation at H_2_O_2_ concentrations of 250 μM, while the former still yielded stochiometric amounts of product at 500 μM concentrations. Inactivation of *Nc*AA9C was only observed at an H_2_O_2_ concentration of 1000 μM.^2^ A more sophisticated method is to determine the average number of peroxidase reactions before inactivation (*n*_max_) or the probability of inactivation (*p*_i_), which is the reciprocal of *n*_max_ in the absence of substrate.^4^ In the study, two AA9-type (fungal) and two AA10-type (bacterial) LPMOs were tested using two different reductants. Interestingly, inactivation was independent of the reductant, but the fungal LPMOs had higher stability turning over more than, i.e., 100 ascorbic acid molecules, while the bacterial enzymes were inactivated between 10 to 28 turnovers. Still, the half-lives of the four enzymes were similar as the fungal LPMOs display higher catalytic efficiencies toward the reductant peroxidase reaction than the bacterial ones.

It is evident that under simplified laboratory conditions, the peroxygenase reaction is preferred by LPMOs. Thus, it was interesting to see that the same is true under physiological conditions on the natural substrate, poplar wood.^5^ Chang et al. showed that if an H_2_O_2_-producing cellobiose dehydrogenase from *Crassicarpon hotsonii* (*Ch*CDH) was brought on the surface of a poplar wood slice, H_2_O_2_ was produced at a rate of 0.85 μM*min^–1^. Then, *Nc*AA9C and its natural electron donor *Nc*CDH were added, and the concentration of H_2_O_2_ and O_2_ was monitored using a piezo-controlled H_2_O_2_ microsensor. Immediately, the H_2_O_2_ concentration decreased, while the O_2_ concentration remained constant showing the preference of LPMO for H_2_O_2_ in the presence of O_2_ on a natural substrate.

Combined, these studies show that the LPMO mechanism is complex. Yet, by carefully designing experiments, important information on the individual pathways can be obtained. Moreover, the studies underscore the significance of the nature of the reductant and that the behavior of LPMOs in certain reactions varies between families. Finally, all the studies demonstrate a clear preference for an LPMO-based peroxygenase reaction spanning soluble, insoluble, as well as intact plant cell wall substrates, and the LPMOs are stable peroxygenases when care is used with respect to the conditions of the experiments.
